# Crystal structure of the Lin28-interacting module of human terminal uridylyltransferase that regulates let-7 expression

**DOI:** 10.1038/s41467-019-09966-5

**Published:** 2019-04-29

**Authors:** Seisuke Yamashita, Takashi Nagaike, Kozo Tomita

**Affiliations:** Department of Computational Biology and Medical Sciences, Graduate School of Frontier Sciences, The University of Tokyo, Kashiwa, Chiba 277-8562 Japan

**Keywords:** RNA, miRNAs, RNA modification, X-ray crystallography

## Abstract

Lin28-dependent oligo-uridylylation of precursor let-7 (pre-let-7) by terminal uridylyltransferase 4/7 (TUT4/7) represses let-7 expression by blocking Dicer processing, and regulates cell differentiation and proliferation. The interaction between the Lin28:pre-let-7 complex and the N-terminal Lin28-interacting module (LIM) of TUT4/7 is required for pre-let-7 oligo-uridylylation by the C-terminal catalytic module (CM) of TUT4/7. Here, we report crystallographic and biochemical analyses of the LIM of human TUT4. The LIM consists of the N-terminal Cys2His2-type zinc finger (ZF) and the non-catalytic nucleotidyltransferase domain (nc-NTD). The ZF of LIM adopts a distinct structural domain, and its structure is homologous to those of double-stranded RNA binding zinc fingers. The interaction between the ZF and pre-let-7 stabilizes the Lin28:pre-let-7:TUT4 ternary complex, and enhances the oligo-uridylylation reaction by the CM. Thus, the ZF in LIM and the zinc-knuckle in the CM, which interacts with the oligo-uridylylated tail, together facilitate Lin28-dependent pre-let-7 oligo-uridylylation.

## Abstract (web summary)

Terminal uridylyltransferase 4/7 (TUT4/7) binds to Lin28 and modifies let-7 precursor (pre-let-7) to regulate cell differentiation and proliferation. Here the authors report the crystal structure of the N-terminal Lin28-interacting module of TUT4, showing a role of the N-terminal zinc finger domain in stabilizing the Lin28:pre-let-7:TUT4 ternary complex.

## Introduction

Let-7 and its family members are highly conserved from nematode to human, and modulate various cellular processes^1–3^. Let-7 expression is coordinately regulated during development, and it promotes differentiation and represses proliferation of cells. Let-7 functions as a tumor suppressor and its expression is downregulated in many cancer cells^4^.

In cells, let-7 expression is controlled by two modes of uridylylation of the precursor let-7 (pre-let-7) in the cytoplasm^5–11^. In differentiated and somatic cells, group II pre-let-7, with a 1-nt 3′-end overhang after Drosha processing, is mono-uridylylated^10,11^. The terminal uridylyltransferase 4 (TUT4) and/or its homolog TUT7 mono-uridylylate group II pre-let-7, and promote subsequent Dicer processing, since pre-let-7 with a 2-nt 3′-overhang is a good Dicer substrate^10^. Thus, TUT4/7 serves as a positive biogenesis factor promoting let-7 expression. In embryonic cells and cancer cells, in which the RNA-binding protein Lin28 is expressed, group I pre-let-7 with a 2-nt 3′-overhang and group II pre-let-7 are oligo-uridylylated, and the expression of let-7 is repressed. Lin28 binds to a conserved 5′-GGAG-3′ motif in the terminal loop of pre-let-7. Lin28 binding to pre-let-7 competes with the Dicer processing of pre-let-7, recruits TUT4/7 to the Lin28:pre-let-7 complex, and promotes the oligo-uridylylation of pre-let-7 by TUT4/7. The oligo-uridylylated pre-let-7 is less responsive to the Dicer processing and is degraded by the 3′–5′ exonuclease DIS3L2^12–14^. The oligo-uridylylation of pre-let-7 reduces let-7 expression, leading to tumorigenesis and embryonic stem cell maintenance. In this case, TUT4/7 functions as a negative regulator of let-7 biogenesis. The functional duality of uridylylation—positive and negative biogenesis factors—by TUT4/7 depends on the status of the 3′-end of pre-let-7 after Drosha processing as well as the cell type, and Lin28 and TUT4/7 act as molecular switches in the developmental and pathological transitions observed in cancer.

Lin28 consists of the N-terminal cold shock domain (CSD) and the C-terminal tandem Cys2HisCys-type zinc knuckles (ZKs). The molecular basis of the interaction between Lin28 and pre-let-7 has been well characterized by biochemical and structural studies^15–17^. The CSD binds to the 5′-GXGAY-3′ motif in the terminal stem-loop structure of pre-let-7 (preE), and the ZKs bind to a conserved 5′-GGAG-3′ motif located near the 3′-end of preE^16,18^.

TUT4 and TUT7 are members of the non-canonical terminal nucleotidyltransferase (TENT) family^19,20^ and are multiple-domain enzymes^21^. The C-terminal half of TUT4/7 is responsible for the nucleotidyltransfer reaction, while the N-terminal half is responsible for the interaction with the Lin28:pre-let7 complex^9^. During the Lin28-dependent oligo-uridylylation of pre-let-7 by TUT4/7, Lin28 and pre-let-7 interact with the N-terminal half of TUT4/7. The interaction between TUT4/7 and Lin28 is pre-let-7-dependent, and Lin28 interacts with TUT4/7 through pre-let-7^22^. The ZKs of Lin28 are necessary and sufficient for the ternary interactions between TUT4/7, Lin28, and pre-let-7^23,24^.

In addition to the regulatory role of TUT4/7 in the biogenesis of let-7, TUT4/7 also participates in the polyA-minus histone mRNA decay in a cell cycle-dependent manner and in polyA-plus mRNA degradation^25–28^. TUT4/7 also uridylylates miRNAs and controls their activity^29–31^, and it also uridylylates the Ago-bound pre-miRNAs for quality control of miRNA synthesis^32^. Recent reports have shown that TUT4/7 participates in immune defense against viruses and retrotransposons by uridylylating viral RNAs and Line-1 mRNAs, respectively^33–35^.

A crystallographic analysis of the C-terminal catalytic module (CM) of human TUT7 complexed with RNAs, including double-stranded RNAs mimicking the stem region of pre-let-7 or oligo-uridylic acids, suggested a mechanism for the mono-uridylylation of group II pre-let7 and the involvement of the zinc knuckles (ZKs) within the CM in the oligo-uridylylation of RNAs^23^. In the absence of Lin28, the 3′-end of pre-let-7 with more than a 2-nt 3′-overhang cannot stably interact with the CM. Thus, TUT4/7 favors the mono-uridylylation of group II let-7 and releases the reaction product. In the presence of Lin28, after several UMP additions to the 3′-end of pre-let7, the ZK2 would recognize the single stranded oligo-uridylylated tail and stabilize the tail in the catalytic pocket, for processive and efficient oligo-uridylylation. However, the detailed role of the N-terminal Lin28-interacting module (LIM) of TUT4/7 for the oligo-uridylylation mode at the C-terminal CM has remained enigmatic, due to the absence of structural information about the LIM.

Here, we present the crystal structure of the N-terminal LIM of human TUT4. Our crystallographic and biochemical analyses revealed the molecular mechanism of the Lin28-dependent oligo-uridylylation of pre-let-7. The structure of the LIM of TUT4, presented in this study, also provides advanced structural information for the design of therapeutic cancer drugs to target the LIM of TUT4/7 and regulate the biogenesis of the let-7 family.

## Results

### Structural determination of the LIM of hTUT4

**Fig. 1:**
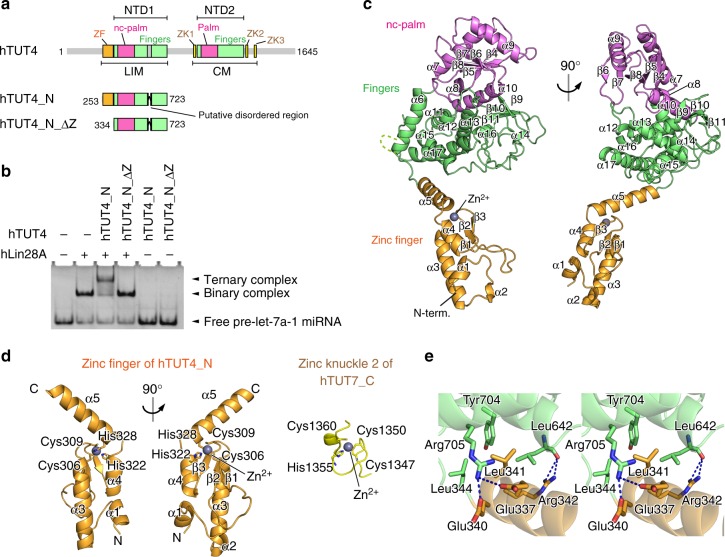
Structure of the Lin28-interacting module of human TUT4. **a** Schematic diagrams of human hTUT4, hTUT4_N, and hTUT4_N_ΔZ. The zinc finger (ZF), the palm, the fingers, and the zinc knuckles (ZK1–3) are colored orange, magenta, green, and yellow, respectively. **b** Gel retardation assays with recombinant hLin28A, hTUT4_N, and hTUT4N_ΔZ in the presence or absence of hLin28A. hTUT4_N, but not hTUT4_N_ΔZ, forms a ternary complex with pre-let-7a-1 and hLin28A. **c** Overall structure of human hTUT4_N, containing the Lin28-interacting module (LIM). The zinc finger (ZF), the non-catalytic palm (nc-palm), and the fingers are colored orange, magenta, and green, respectively, as in **a**. Residues 712–719 are represented by a dashed line. **d** Comparison of the structures of the N-terminal ZF of human TUT4 (left) and the C-terminal zinc knuckle of human TUT7^23^ (right, PDB ID: 5W0N). **e** A detailed view of the interactions between the ZF (orange) and the fingers (green). Hydrogen bonds are depicted by dashed lines

Human TUT4 (hTUT4) is a multi-domain protein consisting of the N-terminal Cys2His2-type zinc finger (ZF), two nucleotidyltransferase domains (NTD1 and NTD2) connected by a flexible linker, and three Cys2HisCys-type zinc knuckles (ZK1–3) in the C-terminal half (Fig. 1a, Supplementary Fig. 1). The NTD1 in the N-terminal half is inactive since it lacks the catalytic carboxylates for the nucleotidyltransferase activity, while the NTD2 in the C-terminal half is responsible for the nucleotidyltransfer reaction. In the presence of pre-let-7, Lin28 interacts with the N-terminal half of TUT4. Hereafter, the N-terminal and C-terminal halves of TUT4 are referred as LIM (Lin28-ineracting module) and CM (catalytic module), respectively^23^ (Fig. 1a).

With an aim toward elucidating the mechanism of the Lin28-dependent oligo-uridylylation of pre-let-7 by hTUT4, we sought to solve the crystal structure of the LIM of hTUT4. To obtain the crystal of the LIM, the N-terminal region (residues 1–252) and an internal putative flexible region (residues 579–610) were truncated from the N-terminal half of hTUT4 (residues 1–723), yielding hTUT4_N (Supplementary Fig. 2). The hTUT4_N contains the ZF and NTD1 and can form a ternary complex with Lin28A and pre-let-7a-1, similar to full-length hTUT4. The deletion of the flexible loop did not affect the ternary complex formation; however, the removal of the N-terminal ZF from hTUT4_N (334–723: hTUT4_N_ΔZ) abolished its ternary complex formation with Lin28A and pre-let-7a-1, suggesting the importance of the ZF for the ternary complex formation (Fig. 1b).

We successfully grew the hTUT4_N crystals. The crystal belongs to the space group *I*2_1_2_1_2_1_ and contains two hTUT4_N molecules in the asymmetric unit. The initial phase was determined by the molecular replacement method using the homology model of the N-terminal NTD1, generated on the basis of the crystal structure of the C-terminal half of hTUT7^23^, as the search model. The structure was refined to an *R* factor of 21.1% (*R*_free_ of 23.8%), using reflections up to 2.4 Å resolution. The final model contains residues 254–711 (except for the truncated residues 579–610), 720–723, and the C-terminal expression tag (residues 724–731) (Fig. 1c, Supplementary Fig. 3). Data collection and refinement statistics are summarized in Table 1.

**Table 1:** Data collection and refinement statistics

	hTUT4_N
Data collection	
Space group	*I*2_1_2_1_2_1_
Cell dimensions	
* a*, *b*, *c* (Å)	113.27, 127.83, 168.80
Wavelength (Å)	0.98000
Resolution (Å)^a^	50–2.4 (2.49–2.40)
*R*_sym_^a^	0.190 (2.322)
*I* /*σI* ^a^	18.1 (1.8)
*CC*_1/2_^a^	0.999 (0.750)
Completeness (%)^a^	99.9 (99.4)
Redundancy^a^	26.8 (26.3)
Refinement	
Resolution (Å)	20–2.4
No. reflections	47,921
*R*_work_ /*R*_free_ (%)	21.07/23.75
No. atoms	
Protein	7134
Ligand	62
Water	90
*B*-factors (Å^2^)	
Protein	70.94
Ligand	76.74
Water	51.31
R.m.s. deviations	
Bond lengths (Å)	0.009
Bond angles (°)	0.97

^a^Values in parentheses are for the highest-resolution shell

### Overall structure of the LIM of hTUT4

The hTUT4_N protein consists of three domains: zinc finger (ZF: residues 253–343), non-catalytic palm (nc-palm; residues 368–489), and fingers (residues 344–367 and 490–723). The nc-palm and the fingers compose the NTD1 (Fig. 1a, c).

**Fig. 2:**
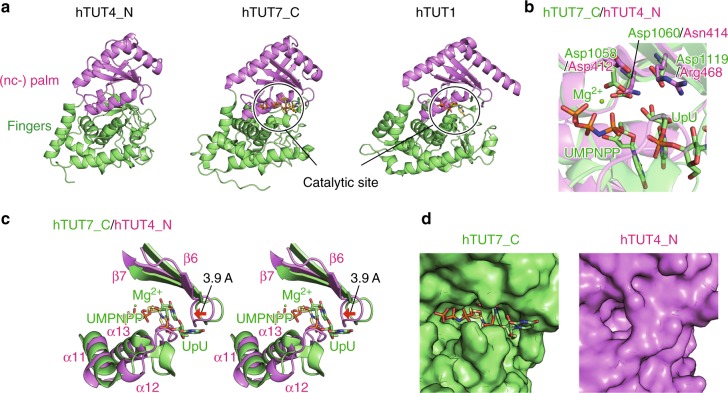
Non-catalytic palm and fingers from the LIM of human TUT4. **a** Comparison of the palm and fingers domains of human TUT4 (amino acid residues 344–723; left) with those of human TUT7_C^23^ (PDB ID: 5W0N, amino acid residues 985–1344; middle) and human TUT1^41^ (PDB ID: 5WU3, amino acid residues 145–599; right). **b** Superimposition of the structures of the catalytic centers of hTUT7_C (green) and hTUT4_N (magenta). The catalytic triad of hTUT7_C and the corresponding residues of hTUT4_N are shown in stick models. **c** Structures of the catalytic pocket of hTUT7_C and the corresponding region of hTUT4_N. **d** Surface models of the catalytic pockets of hTUT7_C (left) and hTUT4_N (right)

The ZF consists of five α-helices (α1–α5) and three β-strands (β1–β3), which compose a large structural domain distinct from other zinc fingers or zinc knuckles, such as the one in the C-terminus of hTUT7^23^ (Fig. 2d). The Cys2His2-type zinc finger motif, in which Cys306, Cys309, His322, and His328 coordinate one zinc ion, forms the core of the ZF. Cys306 and Cys309 reside at the turn of antiparallel β-strands (β2 and β3), and His322 and His328 reside in the following kinked α-helices (α4 and α5). The three α-helices (α1, α2, and α3) and the antiparallel β-strands (β1–β2–β3) support the structure of the core zinc finger motif (Fig. 1d). The ZF is connected to the fingers by the α5 helix (Fig. 1e), which interacts with the fingers. Leu341 forms hydrophobic interactions with Leu344, Leu542, and Tyr704. Glu337 and Glu340 form hydrogen bonds with Arg705, and Arg342 forms a hydrogen bond with the O atom of Leu642. As a result, the orientation and the position of the ZF relative to the NTD1 are fixed.

The overall structure of the NTD1, composed of the nc-palm and fingers, is topologically homologous to those of the catalytic domains of other non-canonical nucleotidyltransferases^36–40^, such as TUT1 and TUT7^23,41^ (Fig. 2a). The nc-palm consists of four α-helices (α7–α10) and five-stranded β-sheets (β4–β8), and is structurally homologous to the catalytic palm of the DNA polymerase β family^42^ (Fig. 2a). The fingers consist of nine α-helices (α6 and α11–α18) and three-stranded β sheets (β9–β11). The putative flexible region, which was truncated for the crystallization, resides between β10 and β11.

A comparison of the structure of the hTUT4 NTD1 with that of the catalytic domain of hTUT7 revealed that two of the three catalytic carboxylates in hTUT7 are substituted with non-catalytic residues in the NDT1 of hTUT4. The three catalytic carboxylates, Asp1058, Asp1060, and Asp1119, in the hTUT7 palm correspond to the Asp412, Asn414, and Arg468 residues in the hTUT4 palm, respectively (Fig. 2b). In addition, a region in the hTUT4 NTD1, which corresponds to the binding pocket of the 3′ end of the primer RNA in the structure of the catalytic domain of hTUT7, is occupied by the loop between α12 and α13, and the loop between β6 and β7 is closer to the cleft between the nc-palm and fingers in the structure of the NTD1 of hTUT4 (Fig. 2c). As a result, the pocket is totally closed, and the 3′-end of the RNA primer and an incoming nucleotide cannot access the region (Fig. 2d). These structural observations confirmed that the NTD1 of hTUT4 is catalytically inactive, and suggested that the NDT1 originated from the active NTD2 through gene duplication and evolved to interact with Lin28 and pre-let-7.

### Involvement of the ZF for the uridylylation of pre-let-7

**Fig. 3:**
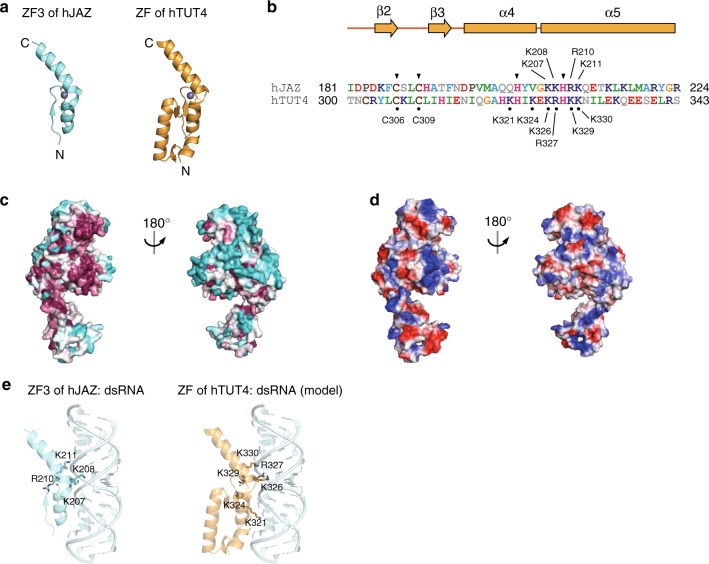
Zinc finger (ZF) of the LIM of human TUT4. **a** Structural similarity between the third zinc finger domain of human JAZ (PDB ID: 2MKN, left)^45^ and the ZF of human TUT4_N (right). **b** Sequence alignment of hJAZ_ZF3 and the ZF of hTUT4_N. The zinc-coordinating residues are indicated by arrowheads. The residues mutated in the biochemical assays are indicated by dots. **c** Conservation analysis of hTUT4_N. Conserved and non-conserved residues are colored purple and cyan, respectively. **d** Electrostatic surface potential of hTUT4_N. The positively and negatively charged regions are colored blue and red, respectively. **e** Docking model of the ZF of hTUT4_N and the double-stranded RNA complex

The ZF of hTUT4 belongs to the U1C type of Cys2His2 zinc finger, based on the number of residues between the cysteine and histidine residues^43^ (C-X_2_-C-X_n_-H-X_5_-H), and is topologically homologous to the third zinc finger of the human double-stranded RNA-binding zinc finger protein JAZ (hJAZ_ZF3, PDB ID: 2MKN). It is also homologous to the zinc finger of yeast tRNA dimethylallyltransferase (PDB ID: 3EPH), in which the zinc finger interacts with the anti-codon helix of tRNA^44^ (Fig. 3a, Supplementary Fig. 4).

In hJAZ_ZF3, the positively charged residues in the helices, which are conserved in hTUT4_N (Fig. 3b), interact with the double-stranded RNA (dsRNA) without sequence specificity^45^. Thus, the ZF of hTUT4_N would also potentially interact with the dsRNA region of pre-let-7. The evolutional conservation analysis and electrostatic surface analysis of the overall structure of hTUT4_N (Fig. 3c, d) revealed that one side of hTUT4_N is highly conserved and positively charged. The area spans from the putative dsRNA binding site in the ZF to the fingers and nc-palm, indicating the involvement of the conserved positively surface in the interaction with the lin28:pre-let-7 complex. A model of the complex of the ZF of hTUT4_N and dsRNA, built based on the structure of the dsRNA-JAZ complex^45^, showed that the positively charged residues on the kinked helices (α4 and α5) would potentially interact with the dsRNA (Fig. 3e).

**Fig. 4:**
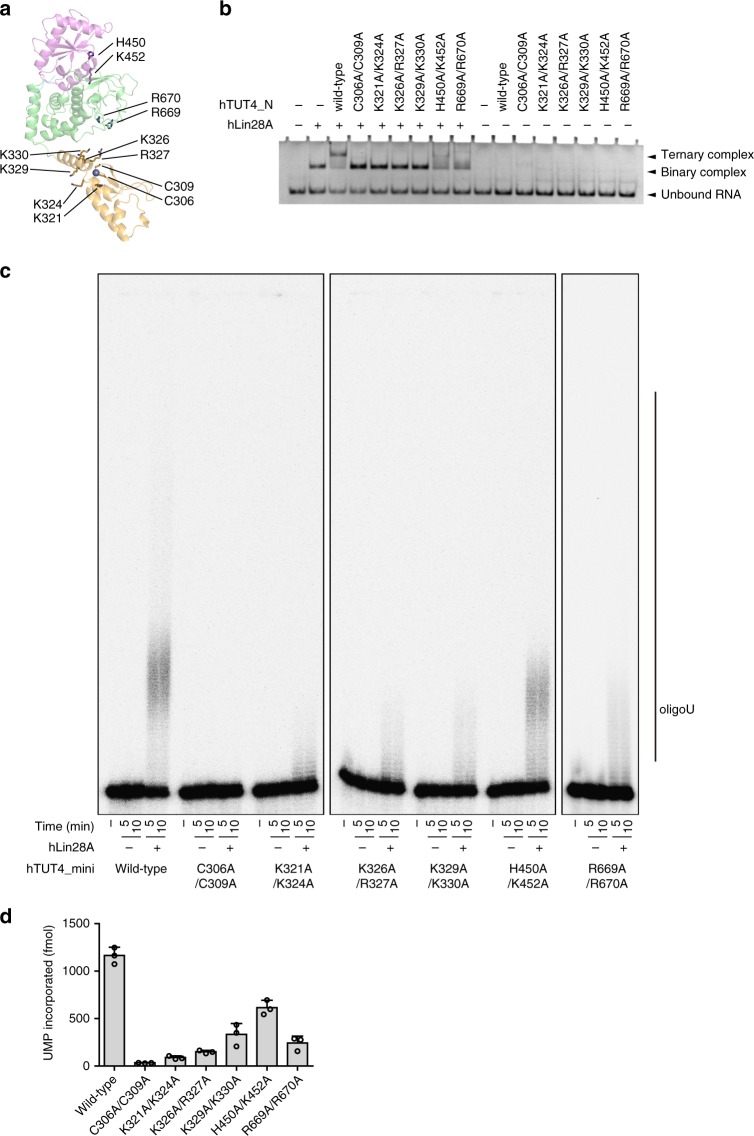
Uridylylation of pre-let-7 by human TUT4 variants. **a** The residues mutated in the LIM for the biochemical analyses in **b** and **c**. **b** Electrophoretic mobility shift of pre-let-7a-1 by wild-type or mutant hTUT4_N proteins, in the presence or absence of hLin28A. The samples were separated by 10% (w/v) polyacrylamide electrophoresis under native conditions, and the gel was stained with ethidium bromide. Experiments were performed three times, and a representative image is shown. **c** In vitro uridylylations of [^32^P]-labeled pre-let-7a-1 by wild-type or mutant hTUT4_mini proteins, in the presence or absence of hLin28A. The reaction products were separated by 10% (w/v) polyacrylamide gel electrophoresis under denaturing conditions. **d** Quantification of α-[^32^P]-UTP incorporation into pre-let-7a-1 by wild-type or mutant hTUT4_mini proteins in the presence of hLin28A, after a 5 min reaction (Supplementary Fig. 6). The bars in the graph are the means ± SD of three independent experiments

The formation of the ternary complex of Lin28:pre-let-7:hTUT4_N is abolished by the truncation of the ZF from hTUT4_N (Fig. 1b), and reduced by the mutations of the conserved residues (Fig. 4a, b, Supplementary Fig. 5). In particular, the mutations in the ZF significantly impact the ternary complex formation. Wild-type hTUT4_N forms a ternary complex with pre-let-7a-1 and Lin28A. However, the C306A/C309A mutations, which disrupt the zinc finger motif in the ZF, abolish the ternary complex formation. The K321A/K324A, K326A/R327A, or K329A/K330A mutations in the ZK also abolish the ternary complex formation. The H450A/K452A or R669A/R670A mutations on the conserved surface of the nc-palm and fingers, respectively, also reduced the ternary complex formation. Consistent with these observations, the hTUT4_mini (residues 202–1313) with the C306A/C309A mutations does not oligo-uridylylate pre-let-7a-1 in the presence of hLin28A, while the wild-type hTUT4_mini oligo-uridylylates pre-let-7a-1 in the presence of hLin28A (Fig. 4c). Other variants of hTUT4_mini have reduced hLin28A-dependent oligo-uridylylating activities on pre-let-7a-1. In the absence of hLin28A, the mono-uridylylation of pre-let7a-1 is not affected by these mutations (Supplementary Fig. 6).

Altogether, the N-terminal ZF of hTUT4 interacts with the dsRNA region of pre-let-7 and contributes to the stable ternary complex formation with Lin28 and pre-let-7, thus leading to the efficient Lin28-dependent oligo-uridylylation of pre-let-7.

### Interactions between Lin28, TUT4_N, and pre-let-7

**Fig. 5:**
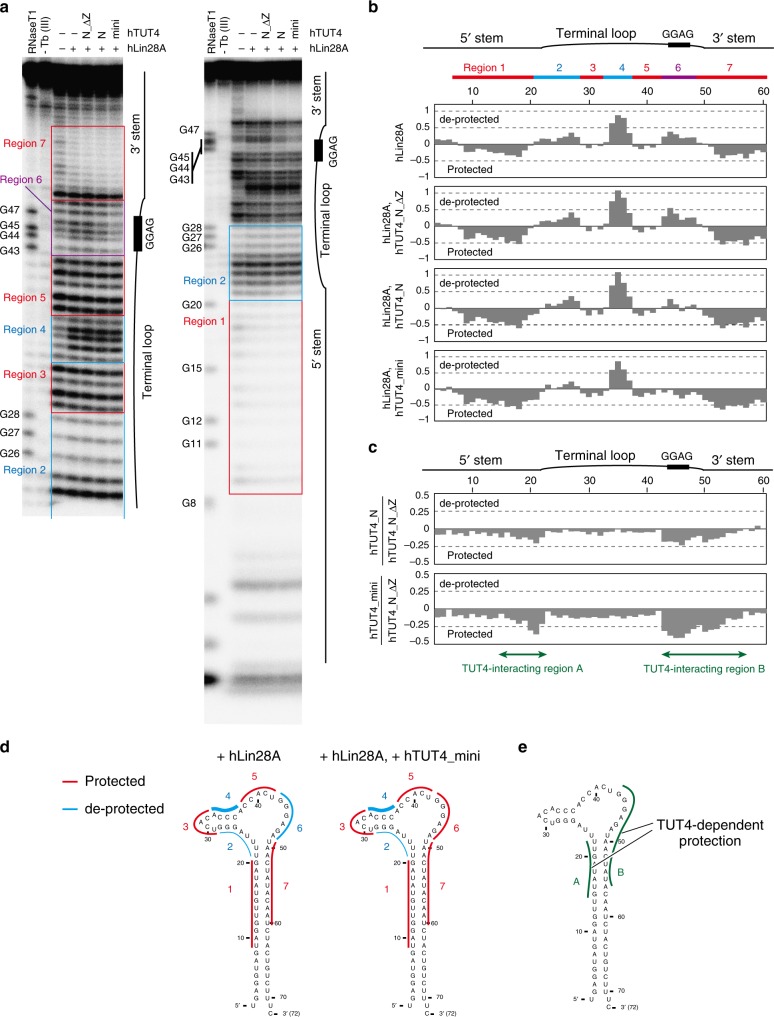
Interactions between Lin28A, pre-let-7a-1, and human TUT4. **a** Tb (III)-mediated hydrolytic pattern of pre-let-7a-1 in the absence and presence of hLin28A and hTUT4 variants. [^32^P]-Labeled pre-let-7a-1 was incubated with Tb (III) in the absence or presence of 1 µM recombinant Lin28A and TUT4 variants. Cleavage patterns were analyzed on 16% (w/v) sequencing gels. Protected and de-protected regions are depicted by red and cyan rectangles, respectively. **b** Protection/de-protection patterns in the presence of hLin28A and hTUT4 variants. Band intensities were quantified and normalized by the cleavage sample without proteins. **c** The protection patterns by the interactions with hTUT4_ N and hTUT4_mini. The ratios of the band intensities in the presence of hTUT4_N and hTUT4_N_ΔZ (upper), and those in the presence of hTUT4_mini and hTUT4_N_ΔZ (lower) were plotted. **d** Superimpositions of the footprinting data of TUT4_N and Lin28A onto the secondary structure of pre-let-7a-1. Protected and de-protected regions are colored as in **a**. **e** Superimpositions of the footprinting data of the TUT4-dependent protection onto the secondary structure of pre-let-7a-1

The interactions between pre-let-7, Lin28, and TUT4 were analyzed by Tb (III) hydrolysis mapping techniques^41,46,47^. The 5′-^32^P-labeled pre-let-7a-1 was incubated with terbium chloride in the presence or absence of Lin28 and TUT4 variants (hTUT4_N_ΔZ, hTUT4_N, and hTUT4_mini), and the protection patterns for the pre-let-7a-1 were assessed (Fig. 5). According to the protection/de-protection patterns, we divided pre-let-7a-1 into seven regions. Regions 1–7 correspond to nucleotides 9–20, 21–28, 29–32, 33–37, 38–42, 43–48, and 49–60, respectively (Fig. 5a).

In the presence of Lin28, regions 2, 4, and 6 were de-protected, and regions 3 and 5 were slightly protected (Fig. 5a, b). Protections at regions 1 and 7 were also observed. These protection/de-protection pattern changes reflect the interactions with Lin28 and the subsequent structural and conformational alterations of the RNA. The crystal structure of Lin28 complexed with the terminal loop of pre-let-7 revealed that the cold shock domain (CSD) and the tandem zinc knuckles (ZKs) interact with the 5′ and 3′ regions of the terminal loop of pre-let-7, respectively^16,48^. Consistent with the structure, Lin28A lacking the CDS showed protection at region 5 and de-protection at region 6, as observed with full-length Lin28A; however, region 4 was not de-protected (Supplementary Fig. 7). The presence of both hLin28A and hTUT4_N_ΔZ did not affect the protection/de-protection pattern of pre-let-7, as compared with the protection/de-protection pattern obtained with hLin28A alone (Fig. 5a, b). This is consistent with the inability of hTUT4_N_ΔZ to form the ternary complex with hLin28A and pre-let-7 (Fig. 1b). In contrast, the presence of hLin28A and hTUT4_N (or hTUT4_mini) yielded further protection (Fig. 5a–d). In particular, region 6 is protected in the presence of both hLin28A and hTUT4_N (or hTUT4_mini), while it is de-protected in the presence of hLin28A alone. Similar protection was observed in the presence of the ZKs of hLin28A and hTUT4_N (Supplementary Fig. 7). The protection/de-protection pattern changes observed in region 6 suggest that the ZKs of hLin28A and hTUT4_N interact with pre-let-7 simultaneously. A comparison of the band intensities in the presence of hLin28A:hTUT4_N_ΔZ and hLin28:hTUT4_N (or hTUT4_mini) revealed that nucleotides 15–22 (region A) and 43–56 (region B) are protected in a hTUT4-dependent manner (Fig. 5e). The hTUT4-dependent overall protection patterns by hTUT4_N and hTUT4_mini are similar to each other, and hTUT4_mini protects more efficiently than hTUT4_N (Fig. 5c). These observations suggested that hTUT4_N and hTUT4_mini both interact with the Lin28:pre-let7 complex, and the C-terminal region of TUT4, the CM, stabilizes and/or enhances the interaction with the Lin28:pre-let-7 complex. Regions A and B include the root of the double-stranded stem, and the 3′ end of the terminal loop containing the 5′-GGAG-3′ motif in pre-let-7 (Fig. 5e).

These results are consistent with the previous report showing that the interactions between the 5′-GGAG-3′ motif in the region 6 of pre-let-7 and the ZKs of Lin28A are the major determinants for the ternary complex formation^23,48^. The enhanced protection at the double-stranded stem region of pre-let-7 also suggests the existence of an interaction between the ZF in hTUT4_N and the double-stranded stem of pre-let-7, and explains the length requirement (~15 bp) of the double-stranded stem of pre-let-7 for the ternary complex formation with Lin28A and TUT4^48^.

## Discussion

**Fig. 6:**
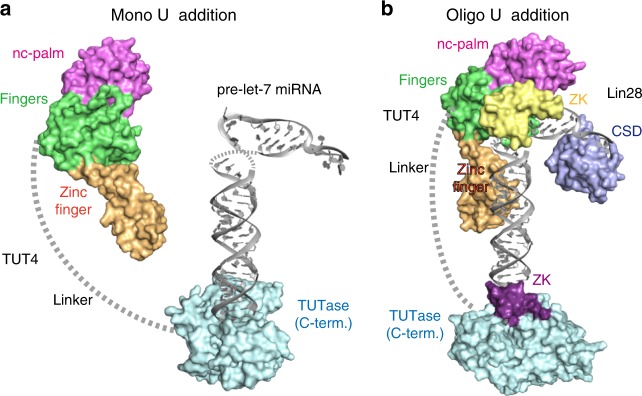
A model of the oligo-uridylylation of pre-let-7 by TUT4. **a** Structural models of the mono-uridylylation of 3’-end of pre-let-7 by human TUT4 in the absence of Lin28 and **b** oligo-uridylylation of the 3′-end of pre-let-7 by human TUT4 in the presence of Lin28. The zinc finger, palm, and fingers domains of hTUT4_N are colored orange, magenta, and green, respectively. The catalytic TUTase domain and the zinc knuckle in the C-terminal half of TUT4 are colored cyan and purple, respectively. The internal linker region is shown by a dotted gray line. The cold shock domain (CSD) and the tandem zinc knuckles (ZK) of Lin28 are colored blue and yellow, respectively

A mechanism for the activity switch between the mono- and oligo-uridylylation of pre-let-7 by TUT4/7 was previously proposed^23^. The transient and unstable interactions between the CM of TUT4/7 and the 3′-end of group II pre-let-7 with a one nucleotide 3′-overhang favor the addition of one uridine and the release of the reaction product before oligo-uridylylation occurs. The 3′-end of the group I pre-let-7 with a 2-nt 3′-overhang and the mono-uridylylated group II pre-let-7 cannot stably bind to the CM of TUT4/7, and thus cannot be substrates for uridylylation (Fig. 6a). Lin28 controls the oligo-uridylylation switch by recruiting TUT4/7 LIM to the 5′-GGAG-3′ motif in the terminal loop of pre-let-7. The stable ternary complex of TUT4/7, Lin28, and pre-let-7 allows the 3′-end of pre-let-7 to relocate into the active site in the CM, and supports the processive oligo-uridylylation by the CM. During the oligo-uridylylation, ZK2 in the CM interacts with the 3′-oligo-uridylylated tail and stabilizes the translocation of the oligo(U) tail. However, the detailed role of the TUT4/7 LIM, in the activity transition from the mono- to oligo-uridylylation of pre-let-7 in the presence of Lin28, has remained unclear, due to the absence of structural information about the LIM.

In this study, we determined the crystal structure of the N-terminal LIM of human TUT4, which consists of ZF and NTD1 (nc-palm and fingers) and is sufficient for the ternary complex formation with Lin28A and pre-let-7. The ternary complex formation is pre-let-7-dependent, and Lin28A interacts with TUT4 through pre-let-7^22^ (Supplementary Fig. 8). The ZF of hTUT4_N adopts a distinct structural domain in the LIM, and its structure is homologous to those of double-stranded RNA-binding zinc fingers (Fig. 4a). The positively charged and conserved surface of the LIM, spanning from the ZF to NTD1, is responsible for the ternary complex formation with pre-let-7 and Lin28. The NTD1 and the N-terminal ZF would potentially interact with the tandem ZKs of Lin28 through the 5’-GGAG-3’ motif of pre-let7, and the double-stranded stem of pre-let7, respectively.

In particular, the ZF plays an important role in the ternary complex formation and the enhancement of the Lin28-dependent oligo-uridylylation in the C-terminal catalytic site (Fig. 6b). After mono-uridylylation of pre-let-7 and translocation of the reaction product, the ZF in the LIM prevents the reaction product from being released from the enzyme. It allows the 3′-end of pre-let-7 to remain and/or relocate in the catalytic pocket of the CM for oligo-uridylylation. The ZK in the CM participates in the stabilization of the oligo-uridylylated tail for processive oligo-uridylylation^23^. Thus, the ZK in the CM and the ZF in the LIM of TUT4 collaboratively facilitate the Lin28-dependent pre-let-7 oligo-uridylylation,

The Lin28/let-7 axis controls various biological processes in cells^1,2,4,9^. The Lin28-dependent pre-let-7 uridylylation, which controls the expression of let-7, is a promising primary therapeutic target. In cancer cells, the reduced expression of let-7 is generally correlated with a poor prognosis. With an aim toward the development of anti-cancer drugs, small molecule compounds that inhibit the Lin28-dependent TUT7 activity have been isolated and other small compounds that block the interaction between Lin28 and let-7 have been reported^49–52^. Thus, the structure of the LIM of TUT4 presented in this study provides structural information for the design of anti-cancer drugs targeting the TUT4/7 activity affecting let-7 biogenesis, by blocking the interactions between TUT4/7 and Lin28.

## Methods

### Expression and purification of recombinant proteins

The sequence encoding human TUT4 (residues 1–1375) was synthesized by Takara Bio (Japan), with codon-optimization for expression in *E. coli*. The nucleotide sequence of the synthetic human TUT4 gene is shown in Supplementary Table 1. For the structural and biochemical assays, DNA fragments encoding hTUT4_N (residues 253–723_Δ579–610) and hTUT4_mini (residues 202–1313) were PCR-amplified and cloned into the *Nde*I and *Xho*I sites of the pET22b and pET29a vectors (Merck Millipore, Japan), respectively. The nucleotide sequences of the primers used for the PCR are shown in Supplementary Table 2. *E. coli* BL21(DE3) (Novagen, Japan) was transformed by the plasmids, and the transformants were grown at 37 °C until the A_600_ reached 1.0. The expression of the TUT4 proteins was induced by adding isopropyl-β-D thiogalactopyranoside at a final concentration of 0.1 mM and incubating the cultures for 18 h at 18 °C. The cells were collected and lysed by sonication in buffer, containing 20 mM Tris-HCl, pH 7.0, 500 mM NaCl, 10 mM β-mercaptoethanol, 20 mM imidazole, 0.1 mM phenylmethylsulfonyl fluoride, and 5% (v/v) glycerol. The proteins were first purified by chromatography on a Ni-NTA agarose column (QIAGEN, Japan), and then further purified on a HiTrap Heparin column (GE Healthcare, Japan). Finally, the proteins were purified by chromatography on a HiLoad 16/60 Superdex 200 column (GE Healthcare, Japan), in buffer containing 20 mM Tris-HCl, pH 7.0, 200 mM NaCl, and 10 mM β-mercaptoethanol. The purified proteins were concentrated and stored at −80 °C until use. The hTUT4 variants were purified in a similar manner.

The sequence encoding human Lin28A (residues 1–209) was purchased from GeneCopoeia. The cDNA was cloned into the *Nde*I and *Xho*I sites of the pET28b vector (Merck Millipore, Japan). The recombinant hLin28A was expressed in *E. coli* Rosetta (DE3) (Novagen, Japan), and purified as described, with the additional purification step of passage through a HiTrap Q column (GE Healthcare, Japan) after the Ni-purification step.

### Crystallization and structural determination of TUT4_N

The crystal used for the structural determination was prepared by the sitting drop vapor diffusion method at 4 °C. The protein concentration was adjusted to 2 mg mL^−1^ with supplementation of 10 mM dithiothreitol (DTT) and 50 µM zinc acetate. A 300 nl portion of the protein solution was mixed with 200 nl of reservoir solution, containing 100 mM Hepes-NaOH, pH 7.0, 20% (w/v) PEG3350, 3% (v/v) 2-methyl-2,4-pentanediol (MPD), 200 mM ammonium citrate, and 3% (w/v) 1,5-diaminopentane dihydrochloride. To facilitate the crystallization, 100 nl of crystal seed solution, prepared with Seed Bead (Hampton Research), was supplemented.

The data set was collected at beamline 17 A at the Photon Factory at KEK, Japan. The crystal was flash-cooled with the reservoir solution supplemented with 20% (v/v) ethylene glycol. The data were indexed, integrated, and scaled with XDS^53^. The initial phase was determined by molecular replacement with Phaser^54^, using the homology model based on the crystal structure of the C-terminal region of human TUT7. The homology model was built by the SWISS-MODEL server^55^. The structure was refined with phenix.refine^56^ and manually modified with Coot^57^. A representative image of the electron density is shown in Supplementary Fig. 3.

### In vitro nucleotide transferase assay

RNA substrates (Supplementary Table 2) were purchased from GeneDesign, Japan. The RNA was phosphorylated before use by ^32^P-labeled or unlabeled ATP, using T4 polynucleotide kinase (Toyobo, Japan). For UMP incorporation into the pre-let-7a-1 RNA, 20 µl reaction mixtures, containing 20 mM Tris-HCl, pH 8.5, 100 mM NaCl, 10 mM MgCl_2_, 10 mM β-mercaptoethanol, 200 µM UTP, 10 nM 5′-[^32^P]-labeled RNA, and 5 nM hTUT4_mini (or its variants), were incubated at 37 °C. At the indicated time points (5, 10 min), a 5 µl portion of the reaction mixture was withdrawn and the reactions were stopped. The ^32^P-labeled RNAs were separated by 10% (w/v) polyacrylamide gel electrophoresis under denaturing conditions. The ^32^P-labeled RNAs were detected with a BAS-5000 or FLA-3000 imager (Fuji Film, Japan). For the experiment with α-[^32^P]-UTP and unlabeled pre-let-7a-1, 10 µl reaction mixtures, containing 20 mM Tris-HCl, pH 8.5, 100 mM NaCl, 10 mM MgCl_2_, 10 mM β-mercaptoethanol, 200 µM unlabeled UTP, 50 nM α-[^32^P]-UTP (3000 Ci/mmole; PerkinElmer, Japan), 250 nM RNA, and 50 nM hTUT4_mini (or its variants), were incubated at 37 °C for 5 min.

### Electrophoretic mobility shift assay

For the electrophoretic mobility shift assays, 300 nM of recombinant hTUT4 protein was incubated with 100 nM pre-let-7a-1, in the presence or absence of 300 nM of recombinant hLin28A, in buffer containing 50 mM Tris-HCl, pH 8.5, 100 mM NaCl, 10 mM MgCl_2_, 10 mM β-mercaptoethanol, and 10% (v/v) glycerol, for 10 min at room temperature. The samples were separated by 6% (w/v) polyacrylamide electrophoresis under native conditions, and the gel was stained with ethidium bromide.

### RNA footprinting with Tb (III)

RNA footprinting with terbium (III) chloride (TbCl_3_) was performed as follows^41,46,47^. The recombinant hTUT4 or its variant proteins were pre-incubated with 100 nM of 5′-[^32^P]-labeled pre-let-7a-1, in 10 μl of buffer containing 50 mM Tris-HCl, pH 8.5, 100 mM NaCl, 10 mM MgCl_2_, 5% (v/v) glycerol, and 10 mM β-mercaptoethanol, for 10 min at 22 °C. Tb (III) was then added to a final concentration of 10 mM. The mixture was incubated for 2 h, and the cleavage reaction was quenched by adding 20 μl of loading buffer, containing 50 mM EDTA, pH 8.0, 0.5% (w/v) sodium dodecyl sulfate (SDS), and 9 M urea. The products were separated by 16% (w/v) polyacrylamide gel electrophoresis under denaturing conditions. The cleavage patterns were analyzed with a BAS-5000 imager, and the band intensities were quantified with the Image Gauge software, Ver. 4.0 (Fuji Film, Japan).

### Immunoprecipitations

To express FLAG-tagged full-length or truncated hTUT4 (Supplementary Fig. 8), the full-length or truncated human TUT4 cDNA was cloned into the pCMV-3Tag1 vector (Agilent, Japan). To express SBP-tagged hLIN28A, the full-length human Lin28A cDNA was cloned into the pSR-V5 vector^58^. HEK293T cells (ATCC, CRL-3216) were grown in DMEM (Wako, Japan) supplemented with 10% fetal bovine serum, at 37 °C in an incubator with a humidified 5% CO_2_ atmosphere. For the immunoprecipitation of SBP-tagged hLin28A, HEK293T cells grown on 10 cm dishes were collected 48 h after transfection of the SBP-hLin28A expression plasmid, together with the FLAG-hTUT4 (or its variants) expression plasmid. The cells were incubated with 1 mL of cell lysis buffer, containing 10 mM Tris-Cl, pH 8.0, 100 mM KCl, 10% (v/v) glycerol, 1% (v/v) Triton X-100, 5 mM DTT, 0.5 mM PMSF, and complete protease cocktail (Roche, Japan). SBP-hLin28A was pulled down from the cell extracts in either the presence or the absence of the pre-let-7a transcript (2 μg), using streptavidin beads (GE Healthcare, Japan). After extensively washing the beads with the cell lysis buffer, SBP-hLin28A was eluted from the beads with 100 μl of the cell lysis buffer containing 2 mM biotin (Sigma, Japan). The eluates were separated on a 4–20% Mini-PROTEAN TGX Precast Protein Gel (Bio-Rad, Japan) and subjected to western blotting using an anti-SBP antibody (Millipore, Japan, catalog number MAB10764) and an anti-Flag antibody (Wako, Japan, catalog number 018–22381). The anti-SBP antibody and the anti-Flag antibody were used at 1:2000 and 1:10,000 dilutions, respectively, for western blotting.

### Computational analysis

The disorder prediction was calculated by the PrDOS server^59^. The surface conservation was analyzed by the Consurf^60^ server.

### Reporting summary

Further information on research design is available in the Nature Research Reporting Summary linked to this article.

## Supplementary information

Supplementary Information

Reporting Summary

Source Data

## Data Availability

A reporting summary for this Article is available as a Supplementary Information file. Coordinates and structure factors have been deposited in the Protein Data Bank, under the accession code 6IW6. The source data underlying Figs. 1b, 4b–d, 5a, and Supplementary Figs. 5b–d, 6, and 7a are provided as a Source Data file. All data are available from the corresponding author upon reasonable request.
