# Supplementary material for: Crystal structure of the Lin28-interacting module of human terminal uridylyltransferase that regulates let-7 expression
Source: Nat Commun. 2019 Apr 29;10:1960. doi: 10.1038/s41467-019-09966-5 (PMC6488673; doi:10.1038/s41467-019-09966-5)
Supplement: Supplementary file 1 — Supplementary Information [file 41467_2019_9966_MOESM1_ESM.pdf]

Supplementary Information

Crystal structure of the Lin28-interacting module of human terminal uridylyltransferase that regulates let-7 expression

Yamashita et al.

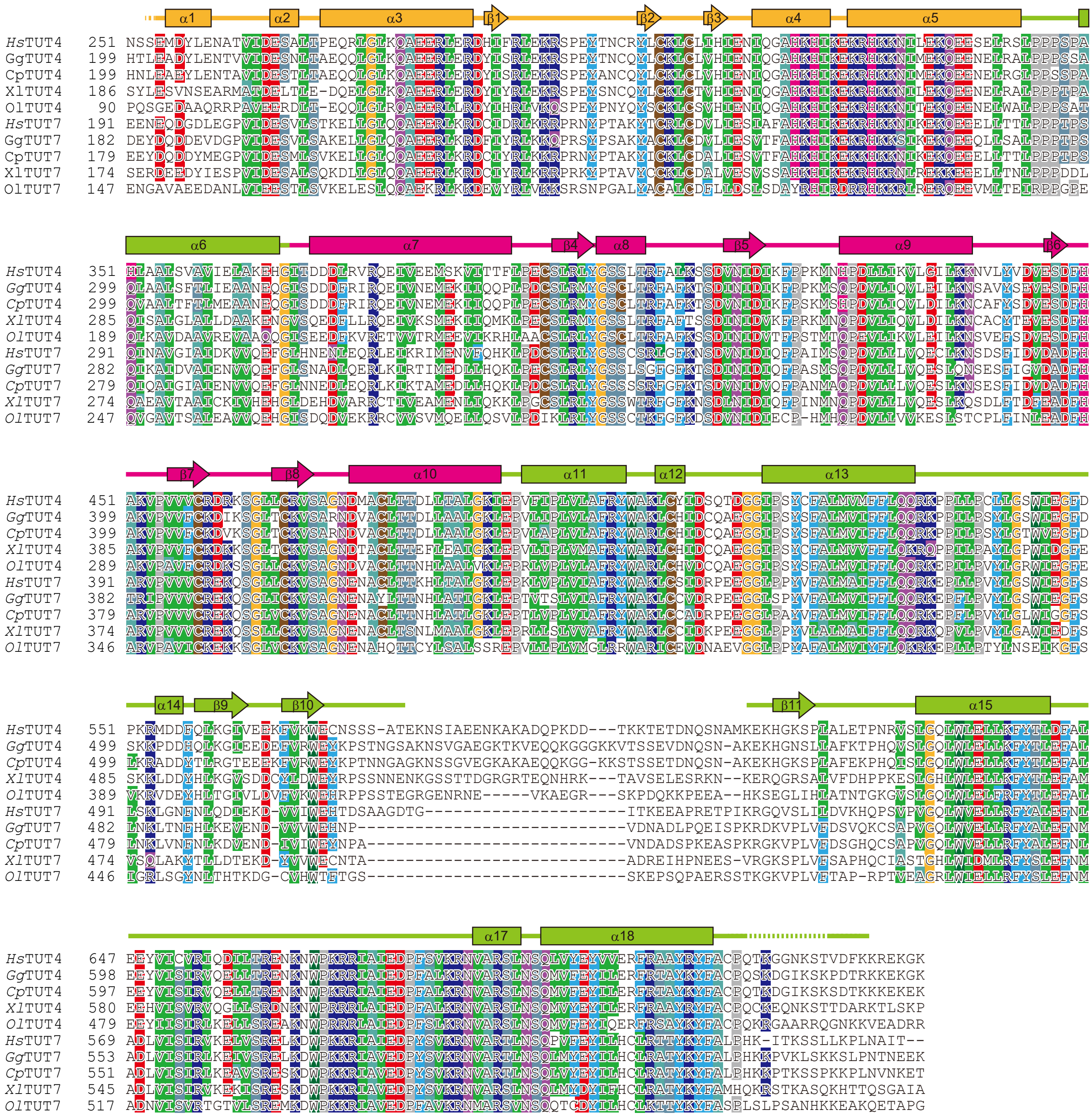

Supplementary Fig. 1: Sequence alignment of the LIMs of TUT4/7 from various organisms. The amino acid sequences of the N-terminal Lin28-interacting module (LIM) of human TUT4 (HsTUT4) and TUT7 (HsTUT7) are aligned with those of the TUT4 or TUT7 proteins from other organisms: *Gallus gallus*, *Chrysemys picta*, *Xenopus laevis*, and *Oryzias latipes*. Secondary structural elements of the LIM of human TUT4, revealed by the crystal structures determined in the present study, are depicted above the sequences, with α-helices and β-sheets shown as rectangles and arrows, respectively. The zinc finger, nc-palm, and fingers are colored orange, magenta, and green, respectively.

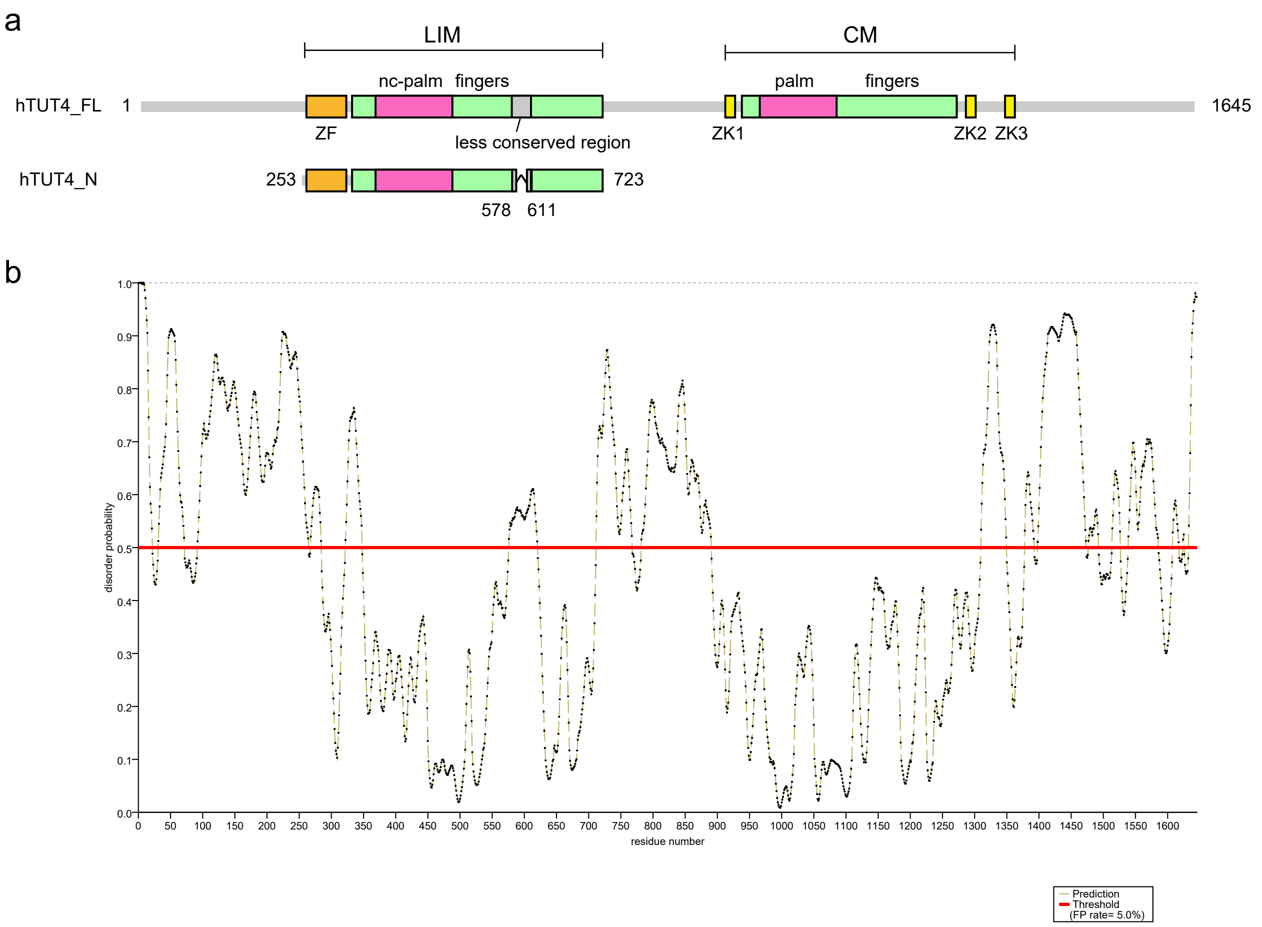

Supplementary Fig. 2: Domain organization and disorder prediction of human TUT4.
(a) Schematic representations of full-length human TUT4 (residues 1-1645; hTUT4_FL) and the truncated N-terminal Lin28-interating module of hTUT4 (residues 253-578, 611-723; hTUT4_N) used for crystallization. The zinc finger (ZF), palm, fingers, and zinc knuckles (ZK) are colored orange, magenta, green, and yellow, respectively. (b) Prediction of natively disordered regions of human TUT4, analyzed by PrDOS (Protein DisOrder prediction System)[1]

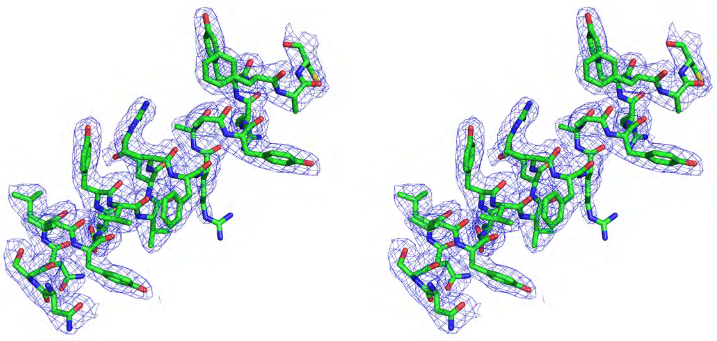

Supplementary Fig. 3: Representative electron density.
Stereo image of the final 2.4 Å 2Fo-Fc electron density map contoured at 1.0 σ around residues Asn688-Cys709 of the LIM of human TUT4.

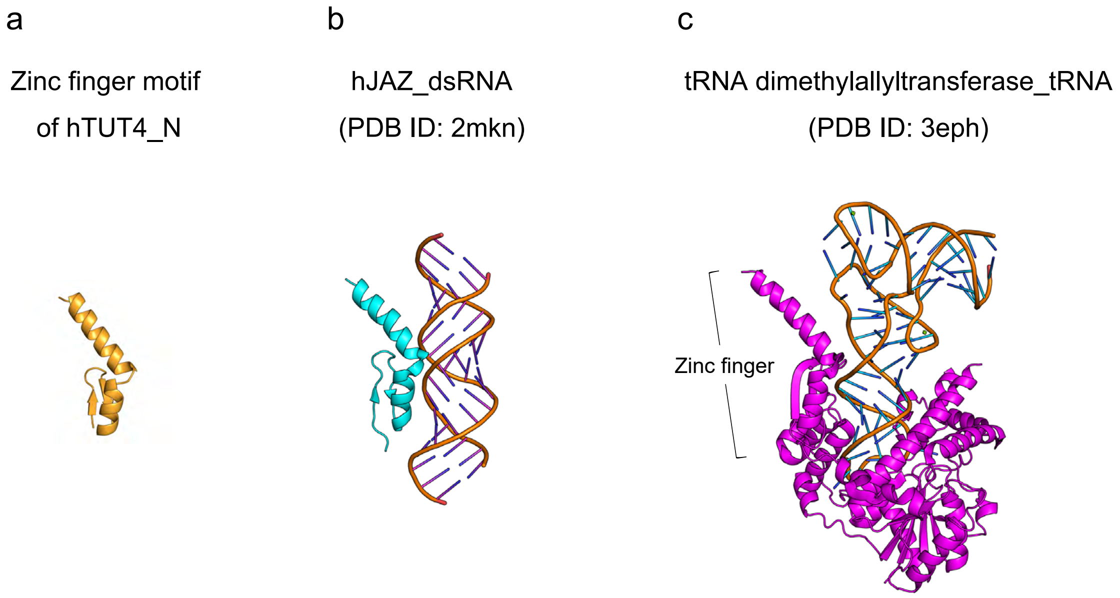

Supplementary Fig. 4: Structures of the "C-X2-C-Xn-H-X5-H" class Cys2His2 zinc finger motif.

(a) Cartoon representation of the zinc finger motif of hTUT4_N. (b) The solution structure of hJAZ bound with double-stranded RNA (PDB ID: 2MKN)[2]. (c) The crystal structure of yeast tRNA dimethylallyltransferase complexed with tRNA (PDB ID: 3EPH). The zinc finger of tRNA dimethylallyltransferase interacts with the anticodon helix of tRNA[3].

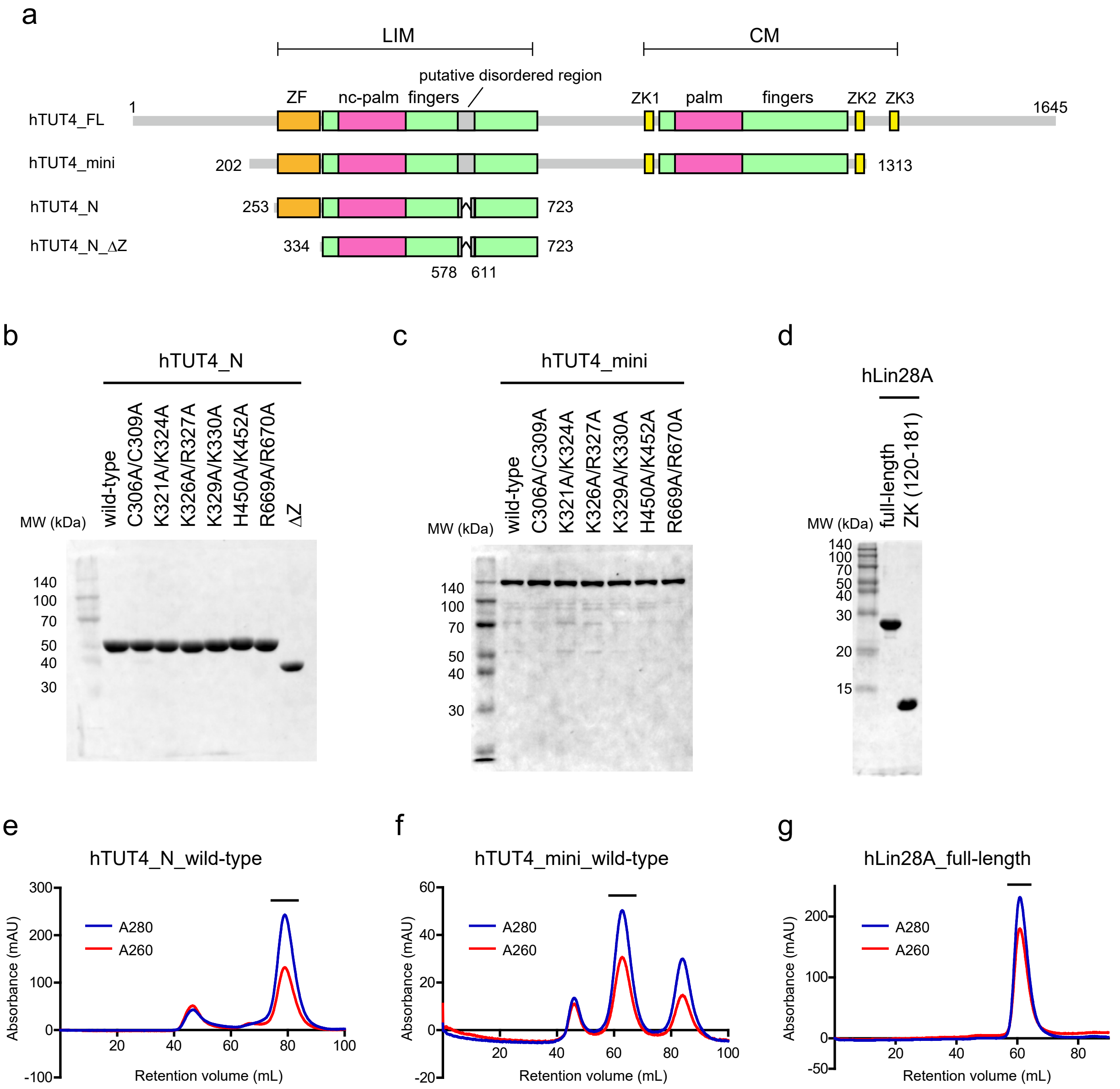

Supplementary Fig. 5: Purification of recombinant human TUT4 variants.
(a) Schematic representations of human full-length TUT4 (hTUT4_FL), hTUT4_mini, hTUT4_N, and hTUT4_N_ΔZ. The zinc finger (ZF), palm, fingers, and zinc knuckles (ZK1-3) are colored orange, magenta, green, and yellow, respectively. (b) SDS PAGE analysis of purified hTUT4_N and its variants. (c) SDS PAGE analysis of purified hTUT4_mini and its variants. (d) SDS PAGE analysis of purified hLin28A and its variants. (e)-(g) Size exclusion chromatography profiles of hTUT4_N, hTUT4_mini, and hLin28A at the final purification steps. The peak fractions highlighted with bars on the graphs were collected and concentrated.

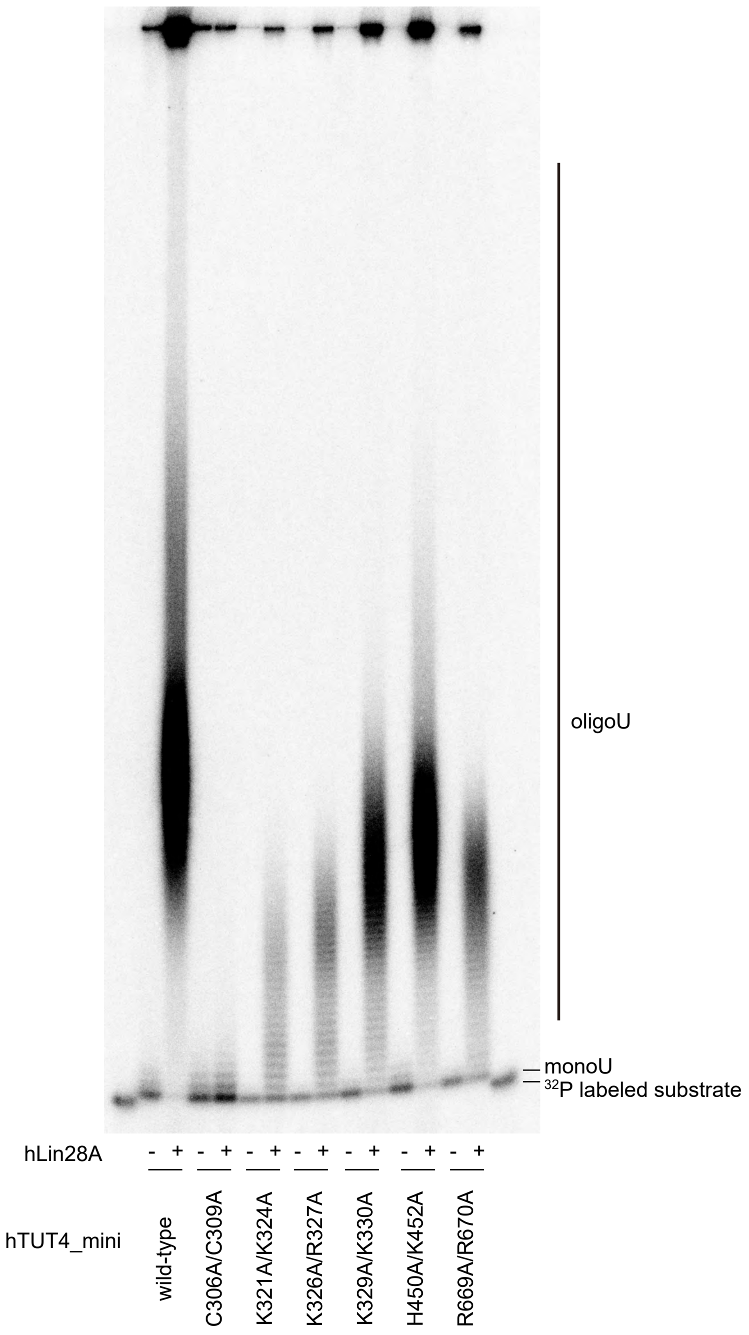

Supplementary Fig. 6: *In vitro* uridylylations of pre-let-7a-1 by hTUT4_mini and its variants. Pre-let-7a-1 was incubated with hTUT4_mini or its variants in the absence or presence of hLin28A, for 5 min using α-[^{32}P]-UTP as a substrate. The reaction products were separated by 10% (w/v) polyacrylamide gel electrophoresis under denaturing conditions and visualized with an imaging plate.

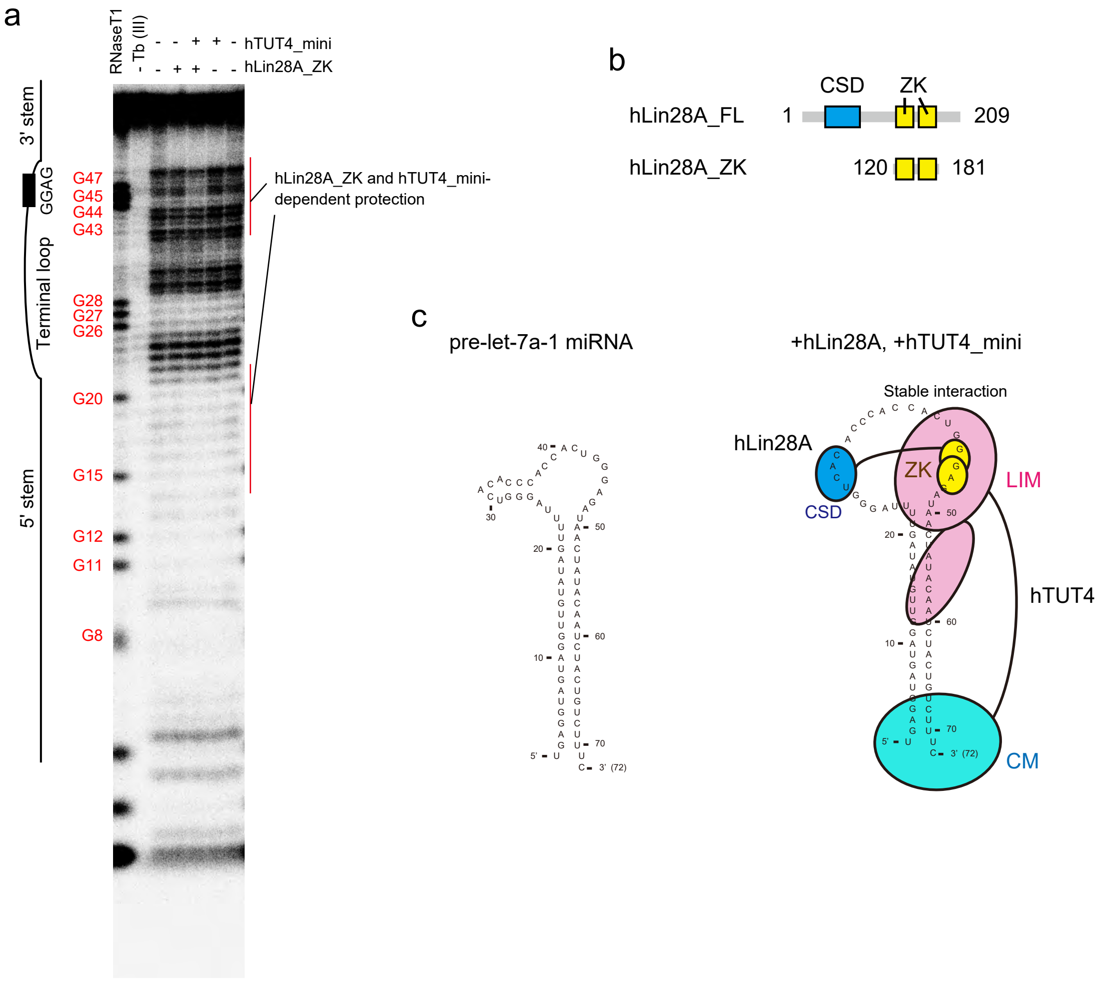

Supplementary Fig. 7: Tb (III) hydrolysis mapping of the binding site of the zinc knuckles of hLin28A and hTUT4_mini to pre-let-7.

(a) Tb (III) hydrolysis in the presence of truncated hLin28A (residues 120-181; hLin28A_ZK) alone and in the presence of both hLin28A_ZK and hTUT4_mini. (b) Schematic diagrams of human Lin28A (hLin28A_FL) and the truncated hLin28A (hLin28A_ZK). The cold shock domain (CSD) and the zinc-knuckle (ZK) are colored blue and yellow, respectively. (c) Cartoon representations of the interactions between pre-let-7a-1, hLin28A, and hTUT4_mini. LIM and CM of hTUT4 are colored pink and cyan, respectively.

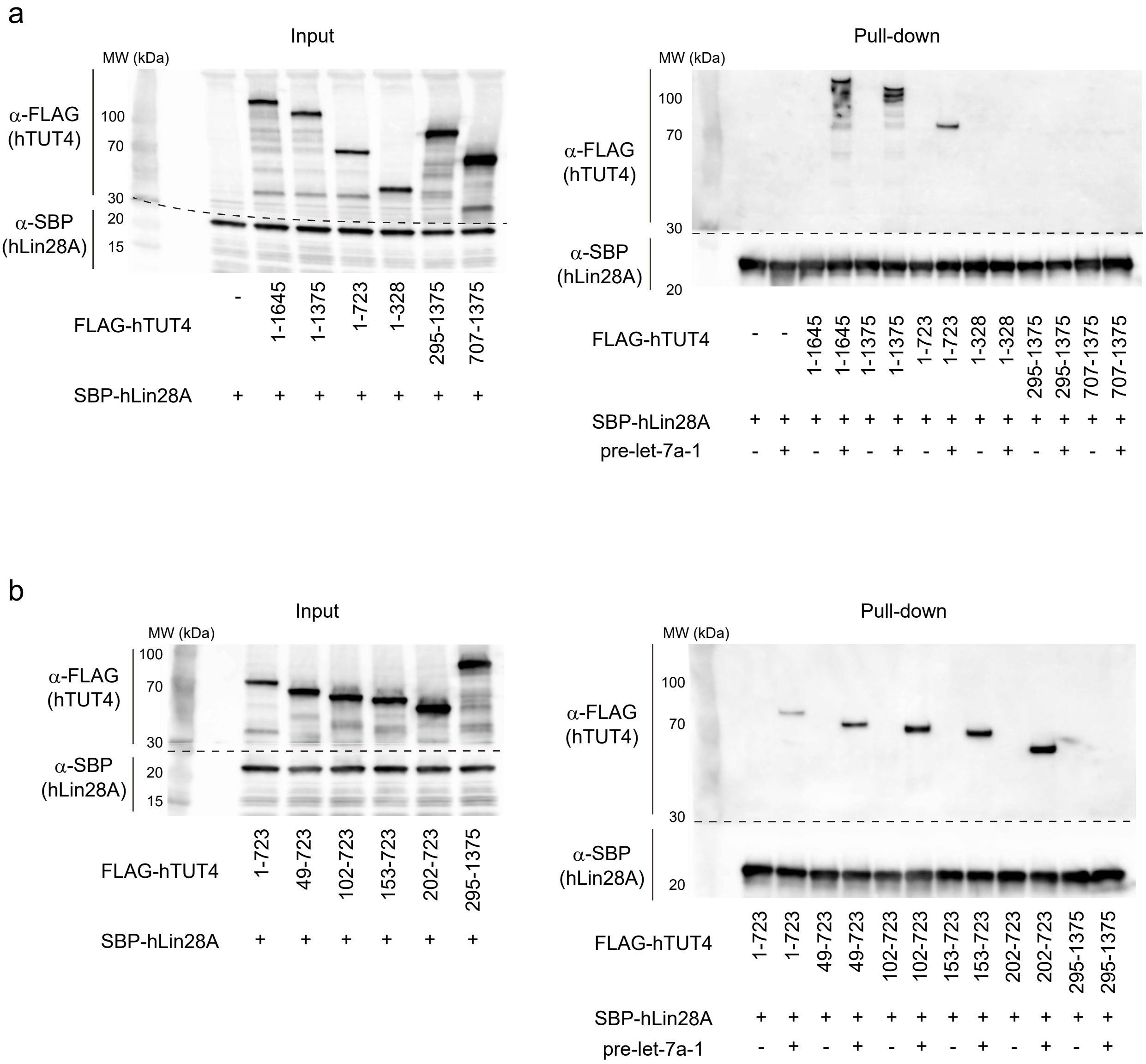

Supplementary Fig. 8: Interactions between Lin28A and the N-terminal half of TUT4.
(a), (b) Lin28A coimmunoprecipitated with the N-terminal half of TUT4, but only in the presence of pre-let-7. FLAG-tagged TUT4 variants and SBP-tagged Lin28A were coexpressed in HEK293T cells. Immunoprecipitation (IP) was performed using anti-SBP antibody-conjugated agarose beads, together with in vitro transcribed pre-let-7a-1. Anti-FLAG and anti-SBP antibodies were used for western blots.

Supplementary Table 1: Nucleotide sequence of the synthetic human TUT4 gene

Human TUT4 coding sequence (amino acid residues 1-1375)	5'-ATGGAAGAAAGCAAAACCCTGAAAAGCGAAAACCATGAACCGAAAAAA<br>AACGTGATTTGCGAAGAAAGCAAAGCGGTGCAGGTGATTGGCAACCAGAC<br>CCTGAAAGCGCGCAACGATAAAAGCGTGAAAGAAATTGAAAACAGCAGCC<br>CGAACCGCAACAGCAGCAAAAAAAACAAACAGAACGATATCTGCATCGAA<br>AAAACCGAAGTGAAAAGCTGCAAAGTGAACGCGGCGAATCTGCCGGGTCC<br>GAAAGATCTGGGCCTGGTTCTGCGCGATCAGAGCCATTGCAAAGCGAAAAA<br>ATTTCCGAACAGCCCGGTGAAAGCGGAAAAAGCGACCATTAGCCAGGCGA<br>AAAGCGAAAAAGCGACCAGCCTGCAGGCGAAAGCGGAAAAAAGCCCGAA<br>AAGCCCGAACAGCGTGAAAGCCGAAAAAGCGTCGAGCTATCAGATGAAAA<br>GCGAAAAAGTGCCGAGCAGCCCGGCAGAAGCGGAAAAAGGTCCGAGCCT<br>GCTGCTGAAAGATATGCGCCAGAAAACCGAACTGCAGCAGATTGGCAAAA<br>AAATTCCGAGCAGCTTTACCAGCGTGGATAAAGTGAACATTGAAGCGGTGG<br>GCGGCGAAAAATGCGCGCTGCAGAACAGCCCGCGCAGCCAGAAACAGCAG<br>ACCTGCACCGATAACACCGGCGATAGCGATGATAGCGCGAGCGGCATTGAA<br>GATGTGAGCGATGATCTGAGCAAAATGAAAAACGATGAAAGCAACAAAGA<br>AAACAGCAGCGAAATGGATTATCTGGAAAACGCGACCGTGATTGATGAAAG<br>CGCGCTGACCCCGGAACAGCGTCTGGGTCTGAAACAGGCGGAAGAACGCC<br>TGGAACGCGATCATATTTTTCGCCTGGAAAAACGCAGCCCGGAATATACCAA<br>CTGCCGCTATCTGTGCAAACTGTGCCTGATTCATATTGAAAACATTCAGGGC<br>GCGCATAAACACATCAAAGAAAAACGCCACAAAAAAAACATCCTGGAAAA<br>ACAGGAAGAAAGCGAACTGCGTAGCCTGCCGCCGCCGAGTCCGGCACATC<br>TGGCAGCACTGTCTGTTGCAGTGATTGAACTGGCGAAAGAACATGGCATTA<br>CCGATGATGATCTGCGCGTGCGCCAGGAAATTGTGGAAGAAATGAGCAAAG<br>TGATTACCACCTTTCTGCCGGAATGCAGCCTGCGCCTGTATGGCAGCAGCCT<br>GACCCGCTTTGCGCTGAAAAGCAGCGATGTGAACATTGATATTAAATTTCCG<br>CCGAAAATGAACCATCCGGATCTGCTGATTAAAGTGCTGGGCATTCTGAAAA<br>AAAACGTGCTGTATGTGGATGTGGAAAGCGATTTTCATGCGAAAGTGCCGG<br>TGGTGGTGTGCCGCGATCGCAAAAGCGGTCTGCTGTGTCGCGTGAGCGCGG<br>GCAATGATATGGCGTGCCTGACCACCGATCTGCTGACCGCGCTGGGCAAAAT<br>TGAACCGGTGTTTATTCCGCTGGTGCTGGCGTTTCGCTATTGGGCGAAACTG<br>TGCTATATTGATAGCCAGACCGATGGCGGCATTCCGAGCTATTGCTTTGCGCT<br>GATGGTGATGTTTTTTCTGCAGCAGCGTAAACCGCCGCTGCTGCCGTGTCTG<br>CTGGGTAGCTGGATTGAAGGCTTTGATCCGAAACGCATGGATGATTTTCAGC<br>TGAAAGGCATTGTGGAAGAAAAATTTGTGAAATGGGAATGCAACAGCAGCA<br>GCGCGACCGAAAAAAACAGCATTGCGGAAGAAAACAAAGCGAAAGCGGAT<br>CAGCCGAAAGATGATACCAAAAAAACCGAAACCGATAACCAGAGCAACGC<br>GATGAAAGAAAAACATGGCAAAAGCCCGCTGGCGCTGGAAACCCCGAATC<br>GCGTTAGCCTGGGTCAACTGTGGCTGGAACTGCTGAAATTTTATACCCTGGA<br>TTTTGCGCTGGAAGAATATGTGATTTGCGTGCGCATTCAGGATATTCTGACCC<br>GCGAAAACAAAAACTGGCCGAAACGCCGCATTGCGATTGAAGATCCGTTTA<br>GCGTGAAACGCAACGTGGCGCGCAGCCTGAACAGCCAGCTGGTGTATGAAT<br>ATGTGGTGGAACGCTTTCGCGCGGCGTATCGCTATTTTGCGTGCCCGCAGAC<br>CAAAGGCGGCAACAAAAGCACCGTGGATTTTAAAAAACGCGAAAAAGGCA<br>AAATCAGCAACAAAAAAACCGGTGAAAAGCAACAACATGGCGACCAACGGC<br>TGCATTCTGCTGGGCGAAACCACCGAAAAAATTAACGCGGAACGCGAACA<br>GCCGGTGCAGTGCGATGAAATGGATTGCACCAGCCAGCGCTGCATTATTGAT<br>AACAACAACCTGCTGGTGAACGAACTGGATTTTGCGGATCATGGCCAGGAT<br>AGCAGCAGCCTGAGCACCAGCAAAAGCAGCGAAATTGAACCGAAACTGGA<br>TAAAAAACAGGATGATCTGGCGCCGAGCGAAACCTGCCTGAAAAAAGAAC<br>TGAGCCAGTGCAACTGCATTGATCTGAGCAAAAGCCCGGATCCGGATAAAA<br>GCACCGGCACCGATTGCCGCAGCAACCTGGAAACCGAAAGCAGCCATCAG<br>AGCGTGTGCACCGATACCAGCGCGACCAGCTGCAACTGCAAAGCGACCGA<br>AGATGCGAGCGATCTGAACGATGATGATAACCTGCCGACCCAGGAACTGTAT<br>TATGTGTTTGATAAATTTATTCTGACCAGCGGCAAACCGCCGACCATTGTGTG<br>CAGCATTTGCAAAAAAGATGGCCATAGCAAAAACGATTGCCCGGAAGATTT<br>TCGCAAAATTGATCTGAAACCGCTGCCGCCGATGACCAACCGCTTTCGCGA<br>AATTCTGGATCTGGTGTGCAAACGCTGCTTTGATGAACTGAGCCCGCCGTGC

	AGCGAACAGCATAACCGCGAACAGATTCTGATTGGCCTGGAAAAATTTATCC<br>AGAAAGAATACGATGAAAAAGCGCGCCTGTGCCTGTTCGGCTCCTCCAAAA<br>ACGGCTTTGGCTTTCGCGATAGCGATCTGGATATTTGCATGACCCTGGAAGG<br>CCATGAAAACGCGGAAAAACTGAACTGCAAAGAAATTATTGAAAACCTGGC<br>GAAAATTCTGAAACGCCATCCGGGCCTGCGCAACATTCTGCCGATTACCACC<br>GCGAAAGTGCCGATTGTGAAATTTGAACATCGCCGCAGCGGCCTGGAAGGC<br>GATATTAGCCTGTATAACACCCTGGCGCAGCATAACACCCGCATGCTGGCGA<br>CCTATGCGGCGATTGATCCGCGCGTGCAGTATCTGGGCTATACCATGAAAGT<br>GTTTGCGAAACGCTGCGATATTGGCGATGCGAGCCGCGGCAGCCTGAGCAG<br>CTATGCGTATATTCTGATGGTGCTGTATTTCCTGCAACAGCGTAAGCCCCCGG<br>TTATTCCGGTGCTGCAGGAAATTTTTGATGGCAAACAGATTCCGCAGCGCAT<br>GGTGGATGGCTGGAACGCGTTTTTTTTCGACAAAACCGAAGAACTGAAAAA<br>ACGCCTGCCGAGCCTGGGCAAAAACACCGAAAGCCTGGGCGAACTGTGGC<br>TGGGCCTGCTGCGCTTTTACACCGAAGAATTCGATTTTAAAGAATACGTGAT<br>CAGCATCCGCCAGAAAAAACTGCTGACCACCTTTGAAAAACAGTGGACCA<br>GCAAATGCATCGCGATCGAAGACCCATTTGATCTGAACCATAACCTGGGCGC<br>GGGCGTGAGCCGCAAAATGACCAACTTTATTATGAAAGCGTTTATTAACGGC<br>CGCAAACTGTTTGGCACCCCGTTTTATCCGCTGATTGGCCGCGAAGCGGAAT<br>ATTTTTTTGATAGCCGCGTGCTGACCGATGGCGAACTGGCGCCGAACGATCG<br>TTGCTGCCGCGTTTGTGGCAAAATTGGCCACTATATGAAAGATTGCCCGAAA<br>CGCAAAAGCAGCCTGCTGTTTCGCCTGAAAAAGAAAGATAGCGAAGAAGA<br>AAAAGAAGGCAACGAGGAAGAAAAGGACAGCCGCGATGTGCTGGATCCGC<br>GCGATCTGCATGATACCCGCGATTTTCGCGATCCGCGCGACCTTCGCTGCTTT<br>ATTTGCGGCGATGCGGGCCATGTGCGCCGCGAATGCCCGGAATAA-3'

Supplementary Table 2: List of synthetic nucleotides

Tut4opt_fromE202_NdeI_Fw	5'-TTTTTTTTCATATGGAAAAATGCGCGCTGCAGAAC-3'
Tut4opt_fromS253_NdeI_Fw	5'-TTTTTTTTCATATGAGCGAAATGGATTATCTGG-3'
Tut4opt_fromE334_NdeI_Fw	5'-TTTTTTTTCATATGGAAAAACAGGAAGAAAGCG-3'
Tut4opt_toK723_nonstop_XhoI_Rv	5'-TTTTCTCGAGTTTAAAATCCACGGTGCTTTTGTTG-3'
TUT4opt_toS1313_nonstop_XhoI_Rv	5'-TTTTCTCGAGGCTTTTGCGTTTCGGGCAATCTTTC-3'
Tut4opt_del579_610_Fw	5'-CAGCAGCAGCGCGACCAAAGAAAAACATGGC-3'
Tut4opt_del579_610_Rv	5'-GCCATGTTTTTCTTTGGTCGCGCTGCTGCTG-3'
TUT4opt_C306A_Rv	5'-CGCCAGATAGCGGCAGTTGGTATATTCC-3'
TUT4opt_C309A_Fw	5'-AAACTGGCGCTGATTCATATTGAAAACATTC-3'
TUT4opt_K321A_Rv	5'-CGCATGCGCGCCCTGAATGTTTTCAATATGAATC-3'
TUT4opt_K324A_Fw	5'-CACATCGCGGAAAAACGCCACAAAAAAAACATCC-3'
TUT4opt_K326A_Rv	5'-CGCTTCTTTGATGTGTTTATGCGCGCCCTGAATG-3'
TUT4opt_R327A_Fw	5'-GCGCACAAAAAAAACATCCTGGAAAAACAGG-3'
TUT4opt_K329A_Rv	5'-CGCGTGGCGTTTTTCTTTGATGTGTTTATGC-3'
TUT4opt_K330A_Fw	5'-GCGAACATCCTGGAAAAACAGGAAGAAAGC-3'
TUT4opt_H450A_Rv	5'-CGCAAAATCGCTTTCCACATCCACATACAG-3'
TUT4opt_K452A_Fw	5'-GCGGCGGTGCCGGTGGTGGTGTGCCGCGATCG-3'
TUT4opt_R669A_Rv	5'-CGCTTTCGGCCAGTTTTTGTTTTCGCGGGTCAG-3'
TUT4opt_R670A_Fw	5'-GCGATTGCGATTGAAGATCCGTTTAGCGTG-3'
hLin28A_5ter_NdeI_Fw	5'-CCGCGCGGCAGCCATATGGGCTCCGTGTCCAACC-3'
hLin28A_fromS120_NdeI_Fw	5'-GCGCGGCAGCCATATGAGTGAGAGGCGGCCAAAAG-3'
hLin28A_3ter_stop_XhoI_Rv	5'-GGTGGTGGTGCTCGAGTCAATTCTGTGCCTCCGGG-3'
hLin28A_toG181_stop_XhoI_Rv	5'-GGTGGTGGTGCTCGAGTTAGCCCTGCTGGGCCTTC-3'
pCMVvec_Fw	5'-GGTACCAGGTAAGTGTACCC-3'
TUT4_328R	5'-TTAATGTCGTTTCTCCTTTATATGTTTATGAGC-3'
TUT4_723R	5'-TTACTTGAAATCCACTGTAGACTTATTTCC-3'
TUT4_1375R	5'-TTACTCTGGGCACTCCCTTCGTAC-3'
TUT4_49F	5'-AAGGGGAATTCTCTCCAAATAGGAATAGTAGTAAAAAAAT AAGCAAAATG-3'
TUT4_102F	5'-AAGGGGAATTCCCTAATTCACCGGTGAAAGCCGAAAAG-3'
TUT4_153F	5'-AAGGGGAATTCGTACCAAGTTCACCAGCAGAAGCAG-3'
TUT4_202F	5'-AAGGGGAATTCGAAAAATGTGCTCTGCAAAACTCACCAC-3'
TUT4_295F	5'-AAGGGGAATTCCGATCACCAGAATATACCAATTGTCGG-3'

TUT4_338F	5'-AAGGGGAATTCAGTGAGCTTCGTTCTCTGCCAC-3'
TUT4_707F	5'-AAGGGGAATTCGCCTGTCCTCAGACGAAG-3'
TUT4_1375R_XhoI	5'-CCTTTCTCGAGTTACTCTGGGCACTCCCTTCGTA-3'
TUT4_723R_XhoI	5'-AAAAGGGGCTCGAGTCACTTGAAATCCACTGTAGACTTATT TCC-3'
pSR-F	5'-CTCGAGCTCTAGCTAAGTAATGCAG-3'
pSR-R	5'-TGCAGCTGATATCCCGGGCTTG-3'
Lin28A-F	5'-GGGATATCAGCTGCAATGGGCTCCGTGTCCAACCAG-3'
Lin28A-R	5'-TAGCTAGAGCTCGAGTCAATTCTGTGCCTCCGGGAG-3'
pre-let-7-a1 RNA	5'-UGAGGUAGUAGGUUGUAUAGUUUUAGGGUCACACCCAC CACUGGGAGAUAACUAUACAAUCUACUGUCUUUC-3'

REFERENCES

1. Ishida, T. & Kinoshita, K. PrDOS: prediction of disordered protein regions from amino acid sequence. *Nucleic Acids Research* **35**, W460-W464 (2007).
2. Burge, R.G., Martinez-Yamout, M.A., Dyson, H.J. & Wright, P.E. Structural Characterization of Interactions between the Double-Stranded RNA-Binding Zinc Finger Protein JAZ and Nucleic Acids. *Biochemistry* **53**, 1495-1510 (2014).
3. Zhou, C. & Huang, R.H. Crystallographic snapshots of eukaryotic dimethylallyltransferase acting on tRNA: Insight into tRNA recognition and reaction mechanism. *Proceedings of the National Academy of Sciences of the United States of America* **105**, 16142-16147 (2008).
