# Supplementary material for: Crystal structure of the Lin28-interacting module of human terminal uridylyltransferase that regulates let-7 expression
Source: Nat Commun. 2019 Apr 29;10:1960. doi: 10.1038/s41467-019-09966-5 (PMC6488673; doi:10.1038/s41467-019-09966-5)
Supplement: Supplementary file 2 — Reporting Summary [file 41467_2019_9966_MOESM2_ESM.pdf]

nature research

Corresponding author(s): Kozo TOMITA

Last updated by author(s): Mar 25, 2019

Reporting Summary

Nature Research wishes to improve the reproducibility of the work that we publish. This form provides structure for consistency and transparency in reporting. For further information on Nature Research policies, see Authors & Referees and the Editorial Policy Checklist.

Statistics

For all statistical analyses, confirm that the following items are present in the figure legend, table legend, main text, or Methods section.

n/a	Confirmed	
☐	☒	The exact sample size (*n*) for each experimental group/condition, given as a discrete number and unit of measurement
☐	☒	A statement on whether measurements were taken from distinct samples or whether the same sample was measured repeatedly
☒	☐	The statistical test(s) used AND whether they are one- or two-sided<br>*Only common tests should be described solely by name; describe more complex techniques in the Methods section.*
☒	☐	A description of all covariates tested
☒	☐	A description of any assumptions or corrections, such as tests of normality and adjustment for multiple comparisons
☒	☐	A full description of the statistical parameters including central tendency (e.g. means) or other basic estimates (e.g. regression coefficient) AND variation (e.g. standard deviation) or associated estimates of uncertainty (e.g. confidence intervals)
☒	☐	For null hypothesis testing, the test statistic (e.g. *F*, *t*, *r*) with confidence intervals, effect sizes, degrees of freedom and *P* value noted<br>*Give P values as exact values whenever suitable.*
☒	☐	For Bayesian analysis, information on the choice of priors and Markov chain Monte Carlo settings
☒	☐	For hierarchical and complex designs, identification of the appropriate level for tests and full reporting of outcomes
☒	☐	Estimates of effect sizes (e.g. Cohen's *d*, Pearson's *r*), indicating how they were calculated

Our web collection on statistics for biologists contains articles on many of the points above.

Software and code

Policy information about availability of computer code

Data collection	Protein Data Bank (PDB), NCBI Database, FLA-3000 system, BAS-5000 system, LAS-4000 mini system, SWISS-MODEL server, beamline BL-17A of KEK
Data analysis	XDS, PHENIX, Coot, and Pymol were used for crystal structural analyses.

For manuscripts utilizing custom algorithms or software that are central to the research but not yet described in published literature, software must be made available to editors/reviewers. We strongly encourage code deposition in a community repository (e.g. GitHub). See the Nature Research guidelines for submitting code & software for further information.

Data

Policy information about availability of data

All manuscripts must include a data availability statement. This statement should provide the following information, where applicable:

- Accession codes, unique identifiers, or web links for publicly available datasets
- A list of figures that have associated raw data
- A description of any restrictions on data availability

The data supporting the findings in this study are available from the corresponding author upon reasonable request. Coordinates and structure factors have been deposited in the Protein Data Bank under the accession code 6IW6.

Field-specific reporting

Please select the one below that is the best fit for your research. If you are not sure, read the appropriate sections before making your selection.

☒ Life sciences ☐ Behavioural & social sciences ☐ Ecological, evolutionary & environmental sciences

For a reference copy of the document with all sections, see nature.com/documents/nr-reporting-summary-flat.pdf

Life sciences study design

All studies must disclose on these points even when the disclosure is negative.

Sample size	No statistical method was used to predetermine sample size. But, the sample number used in this study is comparable with standard biochemical experiments.
Data exclusions	No data were excluded.
Replication	All attempts at replication were successful. We described details for each experiment in online method to reproduce all our experiments.
Randomization	The experiments were not randomized because all biochemical experiments do not require any randomization.
Blinding	No blinding was conducted, because blinding would not provide any reliable datasets for our all biochemical experiments.

Reporting for specific materials, systems and methods

We require information from authors about some types of materials, experimental systems and methods used in many studies. Here, indicate whether each material, system or method listed is relevant to your study. If you are not sure if a list item applies to your research, read the appropriate section before selecting a response.

Materials & experimental systems

n/a	Involved in the study	
☐	☒	Antibodies
☐	☒	Eukaryotic cell lines
☒	☐	Palaeontology
☒	☐	Animals and other organisms
☒	☐	Human research participants
☒	☐	Clinical data

Methods

n/a	Involved in the study	
☒	☐	ChIP-seq
☒	☐	Flow cytometry
☒	☐	MRI-based neuroimaging

Antibodies

Antibodies used	An anti-SBP antibody(Millipore, Japan, catalogue number MAB10764) and an anti-FLAG antibody(Wako, Japan, cagalogue number 018-22381) were used for western blotting at 1:2000 and 1:10000 dilutions, respectively.
Validation	These antibodies were validated in our laboratory by western blotting assays for SBP- or FLAG- tagged recombinant proteins.

Eukaryotic cell lines

Policy information about cell lines

Cell line source(s)	HEK293T (ATCC, CRL-3216)
Authentication	The cell line was confirmed by ATCC.
Mycoplasma contamination	The cell line was not tested for mycoplasma contamination.
Commonly misidentified lines (See ICLAC register)	N/A
